# Very rare localization of a retroperitoneal hemangiopericytoma revealed by lumbosciatalgia: A case report

**DOI:** 10.1016/j.ijscr.2018.10.056

**Published:** 2018-10-30

**Authors:** Amine Chhaidar, Skandar Zouari, Ahlem Bdioui, Moncef Mokni, Ali ben Ali

**Affiliations:** a Department of Surgery, Sahloul Hospital, Sousse, Tunisia; b Department of Urology, Sahloul Hospital, Sousse, Tunisia; c Department of Pathology, Farhat Hached University Hospital, Sousse, Tunisia; d Research Lab: Transfer in Technology in Anatomic Pathology (LR12SP08), Tunisia

**Keywords:** Hemangiopericytoma, Retroperitoneal space, Neoplasm, Vascular tissue, Case report

## Abstract

Introduction: Hemangiopericytoma is a rare vascular tumor representing about 1% of all vascular tumors and approximatively 5% of all soft tissues sarcomas that arises from the pericytes of Zimmerman surrounding capillaries and postcapillaries vessels. Retroperitoneal hemangiopericytomas are rare among the localisations as it usually occurs in lower and upper extremities.

Presentation of case: We present a case of 31-year-old man with a well-defined retro peritoneal mass measuring about 105 × 73 × 83 mm at right lower quadrant of the abdomen anterior to right psoas muscle. Intraoperatively, an large mass measuring 10 × 8 cm was found arising from the retro peritoneum. A monobloc excision was performed. The postoperative course was uneventful. Histopathological examination with immune histochemistry revealed that tumoral cells were positive for desmin, CD34, smooth muscular antigen. Final diagnosis of hemangiopericytoma was made. On the last follow up at one year, he was symptom free.

Discussion: Hemangiopericytoma is a rare tumor. It takes origin from pericytes presenting as intervals along the walls of capillaries and post-capillary venules. Retroperitoneal hemangiopericytoma is a rare location. It is often discovered at late course, and can be revealed by various symptoms with the compression of the adjacent organs by the tumor Surgical resection should be considered in symptomatic cases or in case of diagnostic dilemma.

Conclusion: Retroperitoneal hemangiopericytoma can be benign but it should be treated the same way as aggressive tumors. It requires a careful and long term follow up.

## Highlights

• Hemangiopericytoma is a rare vascular tumor representing about 1% of all vascular tumors and approximatively 5% of all soft tissues sarcomas.
• Retroperitoneal hemangiopericytoma is a rare location.
• The radiologic feature suggests the diagnosis but the pathologic findings associated to immune histochemistery remain the only tool to confirm the diagnosis.
• Surgical excision is the most preferred treatment as it helps in relieving the symptoms as well as confirm the diagnosis.

## 1 Introduction

Hemangiopericytoma is a rare vascular tumor representing about 1% of all vascular tumors and approximatively 5% of all soft tissues sarcomas [1] that arise from the pericytes of Zimmerman surrounding capillaries and postcapillaries vessels. Retroperitoneal hemangiopericytomas are rare among the localisations as it usually occurs in lower and upper extremities [2]. The rarity of these tumors makes the understanding for the pathologic radiologic findings difficult although the progresses made in the level of the sensivity of the new imaging techniques. For now, the guidelines in terms of management of retroperitoneal hemangiopericytomas were established on the basis of case reports and case series and some aspects of the treatment still remain unclear especially when it comes to adjuvant treatment following the surgery. We report a case of retroperitoneal hemangiopericytoma in an adult treated successfully by a monobloc excision. This case has been reported in line with the SCARE criteria [3].

## 2 Case description

A 31 years old patient presented with right-sided L5 radiculopathic pain of three months duration. There was no family history or features of neurofibromatosis. The patient did not have other symptoms including nausea, vomiting, bowel habit changes, fever, or weight loss.

**Fig. 1:**
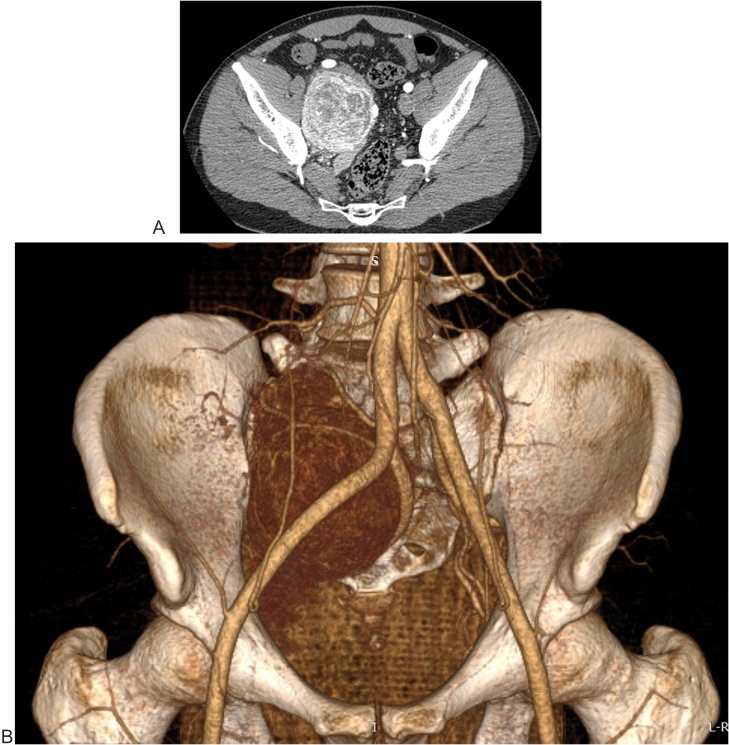
Contrast enhanced computed tomography of abdomen showing a large retroperitoneal mass.

**Fig. 2:**
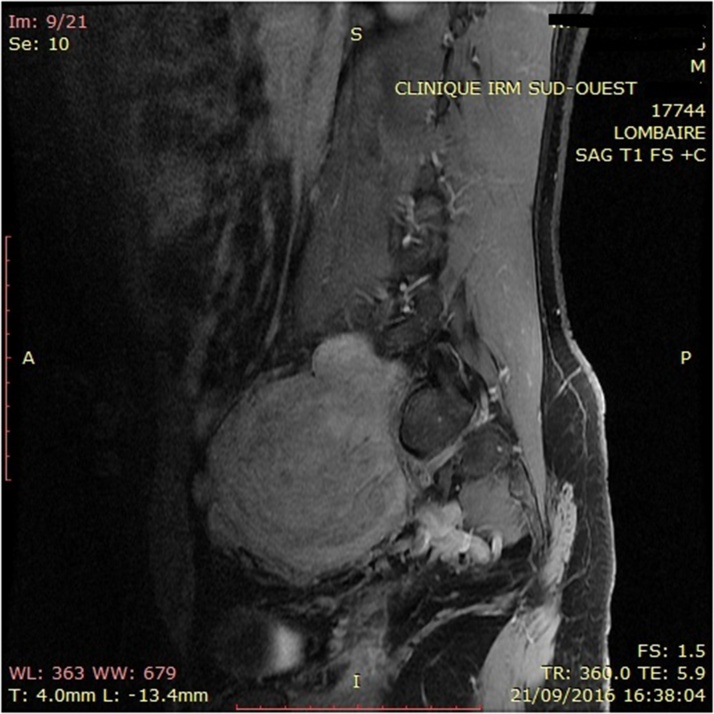
Magnetic Resonance Imaging showing an encapsulated and well limited retro peritoneal masse.

**Fig. 3:**
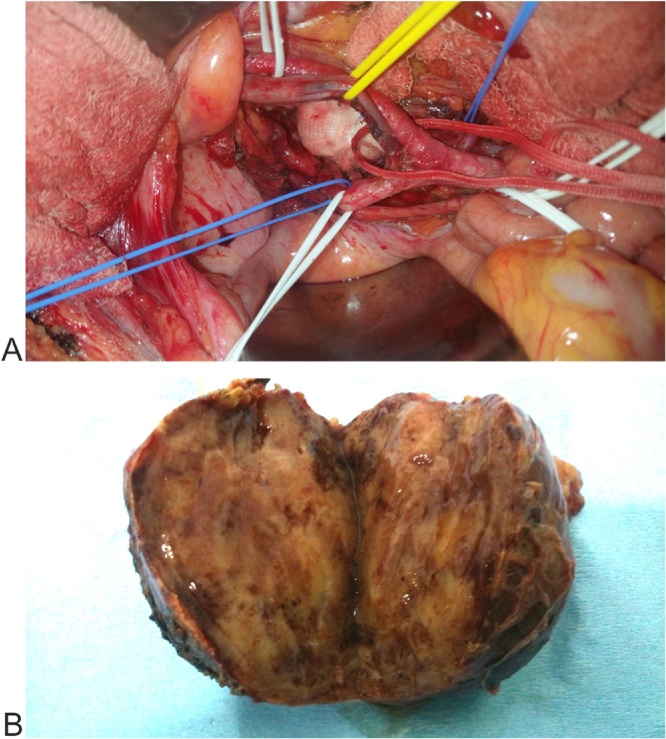
Intraoperative view after mass resection (A) and specimen (B).

**Fig. 4:**
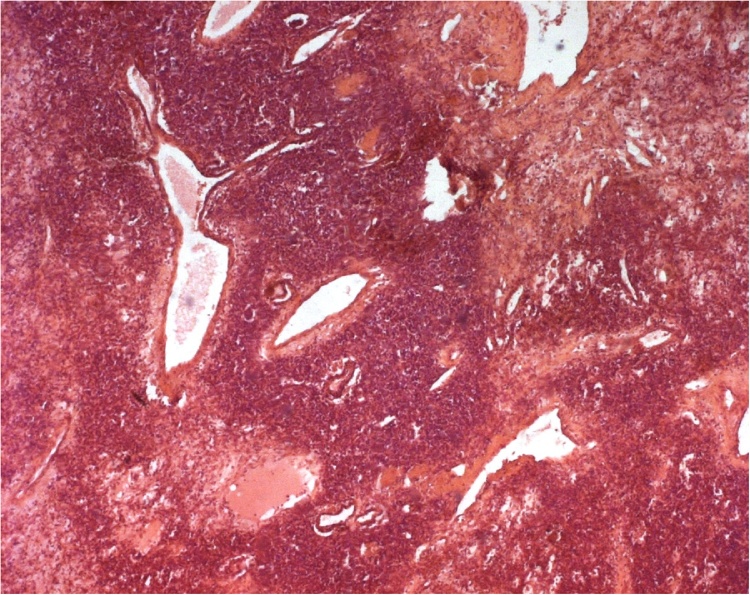
Microscopic examination showing tumor proliferation with variable cell density highly vascularized. (HE x 50).

**Fig. 5:**
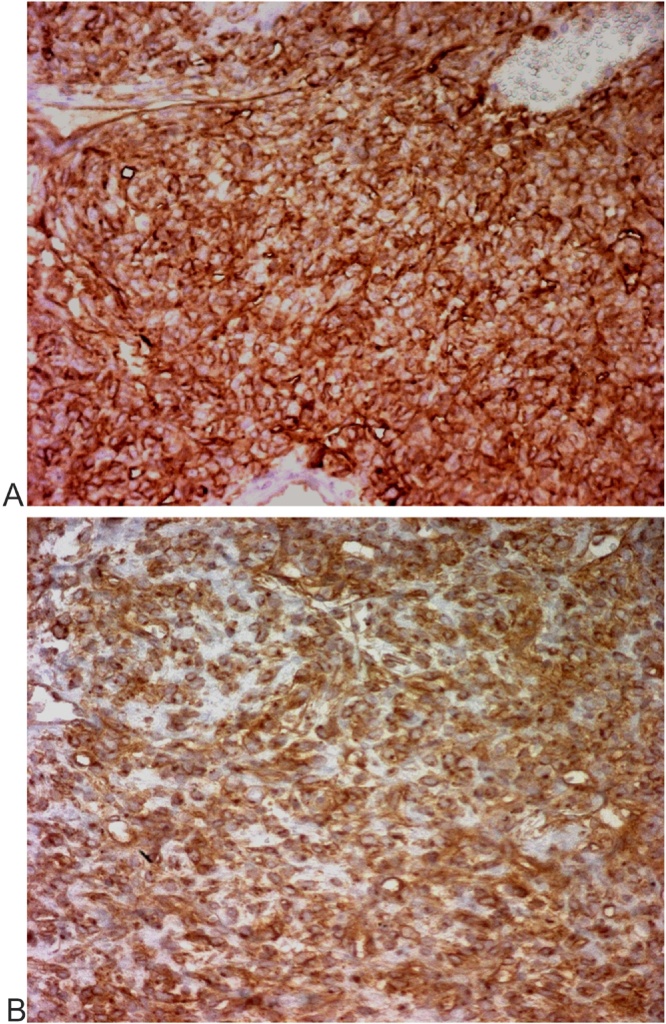
IHC for CD34 - tumor cells strongly positive(A) IHC for CD99 - tumor cells strongly positive (B).

Physical examination showed no positive finding. Computed tomography (CT) showed a well-defined retroperitoneal mass with measuring about 105 × 73 × 83 mm at right lower quadrant of the abdomen anterior to right psoas muscle. After contrast injection, tumoral mass was enhanced intensely (Fig. 1). Magnetic Resonance Imaging showed an encapsulated and well limited retro peritoneal masse occurring in hypo signal T1 and hyper signal T2 with intense enhancement after injection of Gadolinium. It is flush with the right intervertebral foramen L5-S1 (Fig. 2). On exploratory laparatomy, a solitary large retroperitoneal multiloculated mass with hemorrhagic fluid was found (Fig. 3). A monobloc excision was performed (Fig. 3). Further evaluation by immune histochemistry revealed that tumoral cells were positive for desmin, CD34, smooth muscular antigen, and negative for cytokeratin and CD31 (Fig. 4, Fig. 5). Based on these findings, final diagnosis of hemangiopericytoma was made. The patient is under follow up with regular CT scans and after one year, is currently well without any evidence of recurrence.

## 3 Discussion

Hemangiopericytoma is a rare tumor. It takes origin from pericytes presenting as intervals along the walls of capillaries and post-capillary venules and first described by Zimmerman in 1923 [4]. Stout and Murray reported first hemangiopericytoma 20 years later by distinguishing it from glomus tumor [2]. This tumor has a long process of growth. It is discovered when it is large typically presenting as a painless mass, with or without compression of adjacent structures [5]. It has almost equal gender distribution and usually occurs with the fifth decade [6].

Retroperitoneal hemangiopericytoma is a rare location. Enginzer and al. [7] reported an incidence of 24.5% among all hemangiopericytomas. It has a non specific clinical presentation, and occurs as a painless abdominal mass in 75–95%. It is often discovered at late course, and can be revealed by various symptoms with the compression of the adjacent organs by the tumor. Paraneoplasic syndroms have also been reported, consisting on hypoglycaemia, hypertension or gynecomastia [8]. In of the first systematic reviews of hemangiopericytomas [7], describing the clinical features of 96 patients, only two patients had paresthesia as a first symptmatology. In our case, we got an unusual clinical presentation as our patient’s chief complaint was a lumbosciatalgia related to right-sided L5 radiculopathic pain of three months duration.

Plain radiography and ultrasound findings are non specific and in terms of imaging, CT scan is superior to ultrasound, and plays an important role in the predictive diagnosis. They display as well defined lobulated masses, large with speckled calcifications, haemorrhage and areas of necrosis [9]. It is prominently enhanced in double contrast CT scan due to its intense vascularity, especially in the periphery of the tumor (the pseudocapsule), as presented in our case (Fig. 1). This radiologic feature suggests the diagnosis. However, it can occurs in other hypervascularized tumors such as: angiosarcomas, leiomyomas, leiomyosarcomas, schwannomas, mesotheliomas, juvenile hemangiomas, liposarcomas, synovial sarcomas, chondrosarcomas, neuroblastomas and cystic adenoïd carcinomas [10]. MRI also play a key role as it shows a well circumscribed vascularised tumor, and help in the assessment of the boundaries, planes of tumor extension and the relationship to the adjacent viscerias, which is helpful for the preoperative planning [9,11].

But the pathologic findings associated to immune histochemistery remain the only tool to confirm the diagnosis. The pathologic features describe hemangiopericytoma as soft, rubbery with irregular surface. On dissection, extensive haemorrhage and necrosis can be found. Microscopally; we find cells partially or completely enveloped by a basement membrane and basement membrane-like material. These cells express a prominent cytoplasmic filaments; with sometimes interdigitating cytoplasmic processes, and pinocytotic vesicles. Nunnery and al. with a review of 19 cases of hemangiopericytoma found the presence of a basal lamina or basal lamina-like material either partially or completely surrounding tumor cells and separating endothelial cells from pericytes as the most consistent feature [12]. The presence, ultrastructurally, of well-developed basement membrane, myogenic type filaments, and pinocytotic vesicles are highly suggestive of hemangiopericytoma. Immunereactive staining is usually positive for vimentin, smooth muscle actin, muscle-specific actin, factor XIIIa, and VEGF and negative for cytokeratins, factor VIII–related antigen, neuron-specific enolase, KP-1 (CD68), bcl-2, and CD117 (c-*kit*) [13]. McMaster et al. established a classification of the hemangiopericytoma histologically in three categories: benign, borderline malignant, and malignant according to ultrastructural findings like vascular patterns, shape of pericytes, anaplasia of pericytes, number of mitotic figures and reticulum [14]. Preoperative embolization has been described widely with many benefits: it decrease the tumor vascularity, minimize blood loss during resection, control blood supply when attempting the surgery [15]. Authors agree about its therapeutic value in tumor management.

The treatment of choice for this kind of tumor is a wide resection of the whole tumor. Preoperative biopsy of the tumor as well as the enucleation of the tumor are not recommended as the tumor is hypervascularized and do not have a proper capsule [7]. Goldman and al. reported two cases of uncurable hemangiopericytomas due to invasion of the small bowel, omentum, colon or spine [6]. Adjuvant radiotherapy has been used in some cases due to the sensivity of the tumor to radiations, with 45 to 50gr delivered to the patient in 5 weeks [5]. But it is not a common consensus in the literature, and radiotherapy in mainly performed when the tumor is uncurable or the resection incomplete. Remission from radiotherapy alone is rare. Chemotherapy appears to be unuseful in the treatment of curable hemangiopericytomas. Only few cases described short term remission of metastastic tumors using doxorubicin, alone or in combination [16].

In terms of survival, it ranges between 47% and 86% in 10-year survivals with complete resection of the hemangiopericytoma [17]. This difference is due to the nature of the tumor, whether it is malignant or not, especially at the time of the diagnosis. The different items that suggest malignancy of the tumor according to Enzinger and al. [7] are a large tumor with the presence of necrosis, hypercellularity and the presence of more than four mitotic figures per 10 high power fields. Wheras other authors only define malignant tumor by the recurrence of the apparition of metastasis [6,12]. This requires a careful and long term follow up. In our experience; clinical and radiologic follow up by CT Scan is essential to track any sign of recurrence or metastasis. Our patient is regularly seen and for now; has not shown any evidence of recurrence.

## 4 Conclusion

Hemangiopericytoma is today a well defined entity with its clinical; radiologic and especially pathologic characteristics. Large tumors found in the retropeitoneum are usually malignant associated with high mortality rates. Although retroperitoneal hemangioperictyoma can be benign, it should be treated the same way as aggressive tumors; by a wide excision as described in the literature. The literature established in the past the inefficacy of adjuvant radiotherapy; but it may has now a valuable and bigger role in the management of post operative retroperitoneal hemangiopericytoma.

## Conflicts of interest

The authors declare that they have no conflict of interest.

## Funding

This study has not received any funding.

## Ethical approval

The study was approved by Ethics Committee of Hospital Sahloul.

## Consent

Written informed consent was obtained from the patient.

## Author contribution

Amine chhaidar-data collection, Editing of manuscript.

Skandar Zouari– Data collection, Editing of the manuscript.

Ahlem Bdioui – Drafting of manuscript.

Moncef Mokni–Editing of the manuscript.

Ali ben Ali-Editing of the manuscript.

## Registration of research studies

As this was a case report and not a clinical trial, this study does not require registration.

## Guarantor

Amine Chhaidar.

## Provenance and peer review

Not commissioned externally peer reviewed.
